# Load-deformation characteristics of acellular human scalp: assessing tissue grafts from a material testing perspective

**DOI:** 10.1038/s41598-020-75875-z

**Published:** 2020-11-06

**Authors:** Johann Zwirner, Benjamin Ondruschka, Mario Scholze, Gundula Schulze-Tanzil, Niels Hammer

**Affiliations:** 1 Department of Anatomy, University of Otago, Dunedin, New Zealand; 2 Institute of Legal Medicine, University Medical Center Hamburg-Eppendorf, Hamburg, Germany; 3 Institute of Materials Science and Engineering, Chemnitz University of Technology, Chemnitz, Germany; 4 Department of Anatomy and Cell Biology, Paracelsus Medical University, Salzburg and Nuremberg, Nuremberg, Austria; 5 Department of Macroscopic and Clinical Anatomy, Medical University of Graz, Graz, Austria; 6 Department of Orthopaedic and Trauma Surgery, University of Leipzig, Leipzig, Germany; 7 Fraunhofer IWU, Dresden, Germany

**Keywords:** Biomedical engineering, Autologous transplantation, Organ transplantation

## Abstract

Acellular matrices seem promising scaffold materials for soft tissue regeneration. Biomechanical properties of such scaffolds were shown to be closely linked to tissue regeneration and cellular ingrowth. This given study investigated uniaxial load-deformation properties of 34 human acellular scalp samples and compared these to age-matched native tissues as well as acellular dura mater and acellular temporal muscle fascia. As previously observed for human acellular dura mater and temporal muscle fascia, elastic modulus (p = 0.13) and ultimate tensile strength (p = 0.80) of human scalp samples were unaffected by the cell removal. Acellular scalp samples showed a higher strain at maximum force compared to native counterparts (p = 0.02). The direct comparison of acellular scalp to acellular dura mater and temporal muscle fascia revealed a higher elasticity (p < 0.01) and strain at maximum force (p = 0.02), but similar ultimate tensile strength (p = 0.47). Elastic modulus and ultimate tensile strength of acellular scalp decreased with increasing post-mortem interval. The elongation behavior formed the main biomechanical difference between native and acellular human scalp samples with elastic modulus and ultimate tensile strength being similar when comparing the two.

## Introduction

Acellular scaffolds derived from human and animal tissues are utilized increasingly in regenerative medicine to fill tissue defects or experimentally even complete organs^1–3^. The major advantage of these scaffolds is that, in spite being of biological origin, the tissues are exclusively composed of extracellular matrix (ECM), thereby having minimal risk of a host immune response, since antigenic epitopes become broadly removed^4^. An acellular scaffold derived from human skin used as a dura mater replacement has recently been shown to facilitate cellular ingrowth, allowing for both hard and soft tissue regeneration^5^. Moreover, ECM scaffolds facilitate the recruitment of bone marrow-derived progenitor cells and endothelial cells, forming the basis for scaffold vascularization^6^. Load-deformation properties of acellular scaffolds generally lack thorough investigation to date, though such knowledge is crucial for modelling optimized scaffolds capable of facilitating effective tissue regeneration following graft transplantation^7,8^. Moreover, it has been shown that the biomechanical properties of grafts may influence wound healing at the wound edges^7^ following surgical interventions, with the scaffold’s stiffness being a strong regulator of stem cell differentiation^8^. Human acellular skin is a widely deployable graft material, which has, among others, been used for the treatment of chronic wounds^9^, congenital skin absence^10^, tendon repair^11^ or cleft palate repair^5^. This given study is part of the ongoing Human Head Tissues Biomechanics project, which focuses on the mechanical description of human head tissues and their biomechanically adequate surgical replacement. This study aimed at investigating the biomechanical properties of human acellular skin samples from the scalp region under uniaxial tensile loading. Thereby, this study presents baseline data for the future optimization of scalp matrices, providing the best possible structural-mechanical environment for cell ingrowth and scaffold integration. The biomechanical properties of these acellular scalp (AS) scaffolds were compared to native scalp (NS) as well as acellular human dura mater (ADM)^12^ and acellular temporal muscle fascia (ATMF)^13^ as two other representative scaffolds recently investigated by our group. A biomechanical comparison of these scaffolds will facilitate a thorough assessment of potential graft materials for potential new future applications. Based on the previous observations on ADM and ATMF, where the collagenous backbone that is determining the load-deformation behavior of soft tissues was largely unaffected by the acellularization procedure with sodium dodecyl sulphate (SDS) we stated the following hypothesis: the biomechanical parameters of acellular scalp are non-different from native counterparts.

## Material/methods

### Sample retrieval and processing

**Figure 1:**
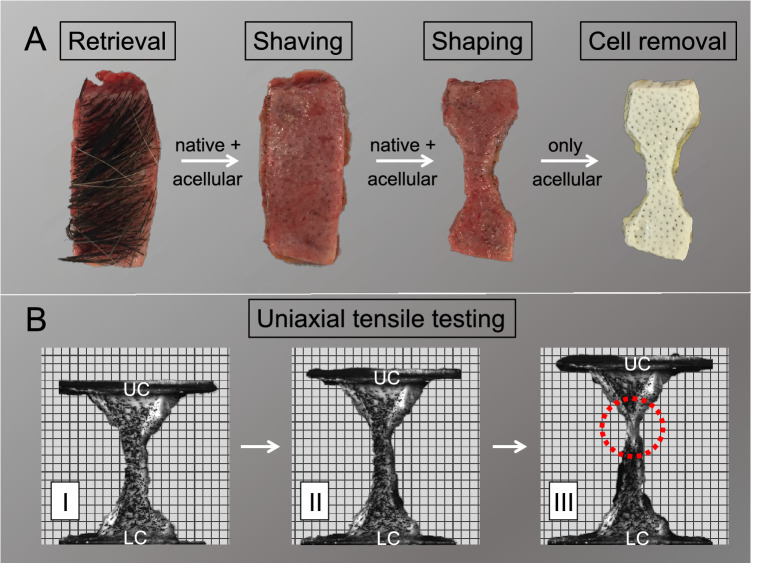
Sample preparation (top row, **A**) and mechanical testing are depicted (bottom row, **B**). (**A)** The samples were shaved off hair and cut into a dog bone shape. Following this, cell removal was conducted for the acellular sample group by means of sodium dodecyl sulphate. (**B)** Dog bone shaped samples were clamped between the upper (UC) and lower (LC) clamps of the material testing machine without load application (I). When loaded, the upper clamp moved away from the lower clamp (II), causing a deformation of the area of parallel measurement length. In the last phase of the experiment, the sample fails in the area of parallel measurement length (red dotted circle) after reaching the maximum force (III).

A total of 68 human scalp samples were harvested at the Institute of Legal Medicine, University of Leipzig, Germany during forensic autopsies. The tissues were evenly distributed into two groups for further processing, an acellular scalp (AS) and a native scalp group (NS). The AS group (13♀, 21♂) had a mean age of 56 ± 22 years (range 17 to 87 years) and a post-mortem interval (PMI) of 77 ± 31 h (range 11 to 120 h). The NS group (13♀, 21♂) had a comparable mean age (range 18 to 87 years) and a PMI of 65 ± 24 h (range 32 to 111 h). The samples were taken from the temporal, fronto-parietal and occipital region. Only macroscopically intact scalp samples without scars and with no known history of systemic dermatologic disorders were selected for this study. The samples were predominantly taken from cadavers that died from sudden events such as an acute myocardial infarction, traumatic brain injury, suicide by hanging, vertebral artery dissection or polytrauma (excluding damage of the used scalp region). The ADM^12^ and ATMF^13^ sample properties for statistical comparison were taken from recent own publications and these data were obtained with similar methods. All methods were carried out in accordance with relevant guidelines and regulations. The protocol has been approved by the Ethics Committee of the University of Leipzig, Germany (protocol number 486/16-ek) and in line with the Saxonian Death and Funeral Act of 1994 (third section, paragraph 18 item 8). As per German law, the state attorney as the legally authorized representative approved the here used tissue samples for research purposes in each individual case. Following the harvesting, the samples were precooled and then transferred to − 80 °C for storage. When defrosted for further processing, the hair was shaved off all samples to assure an even surface for in-plane strain measurements by digital image correlation. Subsequently, the samples were cropped into a dog bone shape by means of a template, adapted from the ISO 527-2 standard (DIC; Fig. 1)^14^. All samples were orientated in an anterior-posterior direction as performed previously^15^ assuring a high level of comparability between studies. Cell removal for the 34 scalp samples of the AS group was performed as described previously^2^. The samples were submerged in an SDS solution of 1 wt.% (Roth, Karlsruhe, Germany) for 7 days. The SDS solution was renewed once after half of the time. Subsequently, the samples were rinsed in distilled water for another 7 days (with daily renewal). Both, SDS submersion and rinsing were performed on a shaking table at a constant temperature of 22 °C.

### Mechanical testing

For the determination of the cross-sectional area, which was required to calculate the ultimate tensile strength (UTS) and the elastic modulus (E_mod_), casts of the samples were produced. Therefore, following the tapering, polysiloxane material (medium-bodied, Exahiflex; GC Corporation, Tokyo, Japan) was used to cast a negative impression of the scalp samples in the parallel measurement area. The resulting casts were scanned at a resolution of 1200-dpi (Perfection 7V750Pro Scanner; Seiko Epson Corporation, Suwa, Japan) with a reference scale before their cross-sectional areas were determined using Measure 2.1d software (DatInf, Tübingen, Germany). To allow for optical strain measurements, a randomly-distributed speckle pattern was created on the samples’ surface using graphite powder. A universal testing machine (AllroundLine Table-top Z020; Zwick Roell, Ulm, Germany) equipped with an Xforce P load cell of 2.5 kN and testControl II measurement electronics (all Zwick Roell) was used to perform uniaxial tensile tests. The upper clamping jaw that was attached to the load cell had a weight of 4.8 kg. This load was the newly defined zero point allowing for non-noisy force readings with the here used load cell even when low forces (< 10 N) were measured. 3D-printed clamps were used, which were recently established to facilitate and standardize soft tissue mechanical testing ^16^. All scalp samples were preconditioned by means of 20 load-unload cycles, applying a force range of 0.5 to 2.0 N. Then, the samples were stretched position-controlled until failure using a crosshead displacement rate of 20 mm/minute and a sampling rate of 100 Hz. The samples deformation was recorded perpendicular to the surface with a DIC system using a single charge-coupled camera enabling for a resolution of 2.8 Megapixels (Q400; Limess, Krefeld, Germany). Strain data of the tensile tests were evaluated by the ISTRA 4D software (VRS 4.4.1.354; Dantec Dynamics, Skovlunde, Denmark).

### Histology, immunolabeling and scanning electron microscopy

Six representative scalp samples (three NS and three AS) were fixed in 10% neutral-buffered paraformaldehyde solution (Sigma-Aldrich, Munich, Germany), dehydrated and subsequently embedded in paraffin. A H&E stain was performed for an overview stain to observe general microscopic changes between native and acellular scalp samples. An Alcian Blue stain was performed to assess the sulphated glycosaminoglycans and a Weigert's Resorcin-fuchsin stain to visualize elastic fibers in native and acellular samples. Moreover, specific immunostains were performed to assess the following components between native and acellular scalp samples: collagen type I and III, fibronectin and decorin. The staining protocol for the aforementioned stains is outlined below. Sections of 7 μm were deparaffinized with xylene and rehydrated in a descending ethanol series. For hematoxylin and eosin (H&E) staining deparaffinized sections were incubated for six minutes in Harry's hematoxylin (Sigma-Aldrich), rinsed in tap water and counterstained for four minutes in eosin (Carl Roth, Karlsruhe, Germany). For the performance of Alcian blue (AB) staining, rehydrated sections were incubated for three minutes in one percent acetic acid and then immersed for 30 min in 1% AB staining solution (Carl Roth). Following the rinsing in 3% acetic acid and a 2 min washing step in distilled water, cell nuclei were counterstained for 5 min using nuclear fast red aluminium sulphate solution (Carl Roth). Rehydrated sections were stained using Weigert's Resorcin-fuchsin (Waldeck GmbH, Münster, Germany) for 5 min to detect elastic fibers. Thereafter, the sections were differentiated in hydrochloric acid/ethanol for 1 min. After washing with tap water for 10 min, an additional washing step in distilled water was conducted. Nuclear fast red aluminum sulphate solution was applied to counterstain the cell nuclei in the Resorcin-fuchsin stains. All stained sections were entellan covered (Merck-Millipore, Darmstadt, Germany). Images were taken using a DM1000 LED light microscope (Leica, Wetzlar, Germany).

For the immunostainings, the above-mentioned samples were washed three times with Tris buffered saline (TBS: 0.05 M Tris, 0.015 M NaCl, pH 7.6), before being incubated with a 0.1% Pronase ready to use solution (BioLogo, Kronshagen, Germany) to demask epitopes for 15 min at 37° C. Subsequently, the sections were re-rinsed with TBS before being incubated with protease-free donkey serum (5% diluted in TBS with 0.1% Triton X 100 for cell permeabilization) for 20 min at room temperature. From here immunostainings for type I/III collagen, decorin and fibronectin were performed as described previously, including counterstaining with 4′,6-diamidino-2-phenylindole (DAPI)^12^. Details of the antibodies used are shown below, (Table 1) Labeled sections were then washed three times for 5 min with TBS before being mounted with Fluoromount mounting medium (Southern Biotech, Eching, Germany) and digitalized using a SPEII confocal laser scanning microscope (Leica).

**Table 1:** The antibodies used for the immunolabelings in this study are depicted.

Target	Primary antibody	Dilution	Secondary antibody	Dilution
Collagen type I	Goat anti human, Abcam, Cambridge, UK	1:50	Donkey anti goat, Alexa Fluor 488, Invitrogen, Carlsbad, USA	1:200
Collagen type III	Mouse anti human, Acris Laboratories	1:30	Donkey-anti-mouse cyanine-3 (cy3), Invitrogen	1:200
Decorin	Rabbit anti human, Acris Laboratories	1:30	Donkey anti rabbit, Alexa Fluor 488, Invitrogen	1:200
Fibronectin	Mouse-anti-human, Dianova, Hamburg, Germany	1:30	Donkey-anti-mouse cy-3, Invitrogen	1:200

Additionally, scanning electron microscopy (SEM) was performed on another two samples (one NS and one AS) by means of a JEOL 6700F field emission scanning electron microscope (JEOL, Peabody, MA, USA). The samples were dehydrated and then coated in a K575X sputter coater with a five-nanometer layer of gold-palladium (Emitech Technologies, Kent, England).

### Data assessment and statistical analysis

The mechanical properties of the scalp samples were evaluated from the DIC data and synchronized force readings using MATLAB R2017b software (Mathworks, Natick, MA, USA). Engineering stress-strain curves were calculated under inclusion of the cross-sectional areas. E_mod_, UTS and strain at maximum force (SF_max_) were assessed. The E_mod_ was determined in the linear region at the beginning of each stress-strain curve and UTS as the maximum stress value of each graph. SF_max_ was set as the corresponding engineering strain when reaching the maximum force or UTS, respectively. Excel Version 16.15 (Microsoft Corporation, Redmond, USA) and GraphPad Prism software version 8 (GraphPad Software, La Jolla, CA, USA) were used for the statistical evaluation. The D’Agostino & Pearson normality test was used to test the Gaussian distribution of the samples. Parametric data of samples were then tested using an ordinary one-way ANOVA. A Kruskal–Wallis test was used for nonparametric data. A Fisher’s LSD (least significant difference) test for ANOVA and uncorrected Dunn’s test for Kruskal–Wallis has been applied. Pearson and Spearman correlation coefficients were reported for normally and non-normally distributed values, respectively. *P* values equal to or smaller than 0.05 were considered statistically significant. Mean values ± standard deviations are reported in the text.

## Results

### Biomechanical parameters of the acellular scalp and its relation to the native scalp and two other acellular graft materials

**Figure 2:**
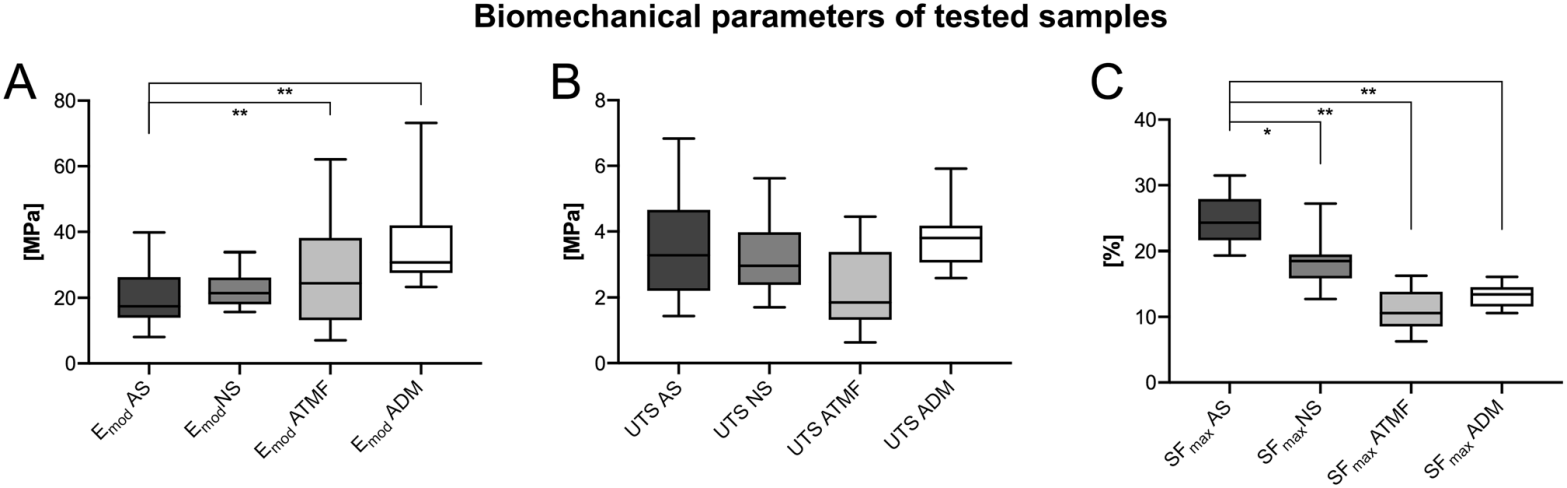
The biomechanical properties of the human acellular scalp (AS) are shown in comparison to the human native scalp (NS), acellular human temporal muscle fascia (ATMF) and human acellular dura mater (ADM). (**A)** Elastic modulus; E_mod_, elastic modulus (in MPa) (**B)** ultimate tensile strength; UTS, ultimate tensile strength (in MPa) (**C)** strain at maximum force; SF_max_, strain at maximum force (in %) The outlines of the boxes indicate the 25% and 75% percentile, the solid black horizontal line the median. Whiskers represent the minimum and maximum. The asterisks indicate significantly different p-values (*, p ≤ 0.05; **, p ≤ 0.01).

Human AS had an averaged E_mod_ of 20.0 ± 9.0 MPa (median = 17.5 MPa) and were statistically non-different from NS (22.5 ± 5.1 MPa, median = 21.4 MPa, p = 0.127; Fig. 2). However, the E_mod_ of AS was significantly different from ATMF (25.7 ± 15.8 MPa, median = 24.5 MPa, p = 0.002) and ADM (35.6 ± 12.4 MPa, median = 30.8 MPa, p = 0.002). The UTS of AS (3.6 ± 1.5 MPa, median = 3.2 MPa) was non-different from NS (3.2 ± 1.0 MPa, median = 3.0 MPa, p = 0.799), ATMF (2.3 ± 1.2 MPa, median = 1.9 MPa, p = 0.469) and ADM (3.8 ± 1.0 MPa, median = 3.8 MPa, p = 0.891; Fig. 2). The SF_max_ of AS (24.7 ± 3.6%, median = 24%) was significantly higher compared to NS (18.3 ± 3.4%, median = 18.5%, p = 0.021), ATMF (11.1 ± 3.1%, median = 10.6%, p < 0.001) and ADM (13.2 ± 1.8%, median = 13.4%, p < 0.001; Fig. 2).

### Elastic modulus and ultimate tensile strength of human acellular scalp decrease with increasing post-mortem interval

**Figure 3:**
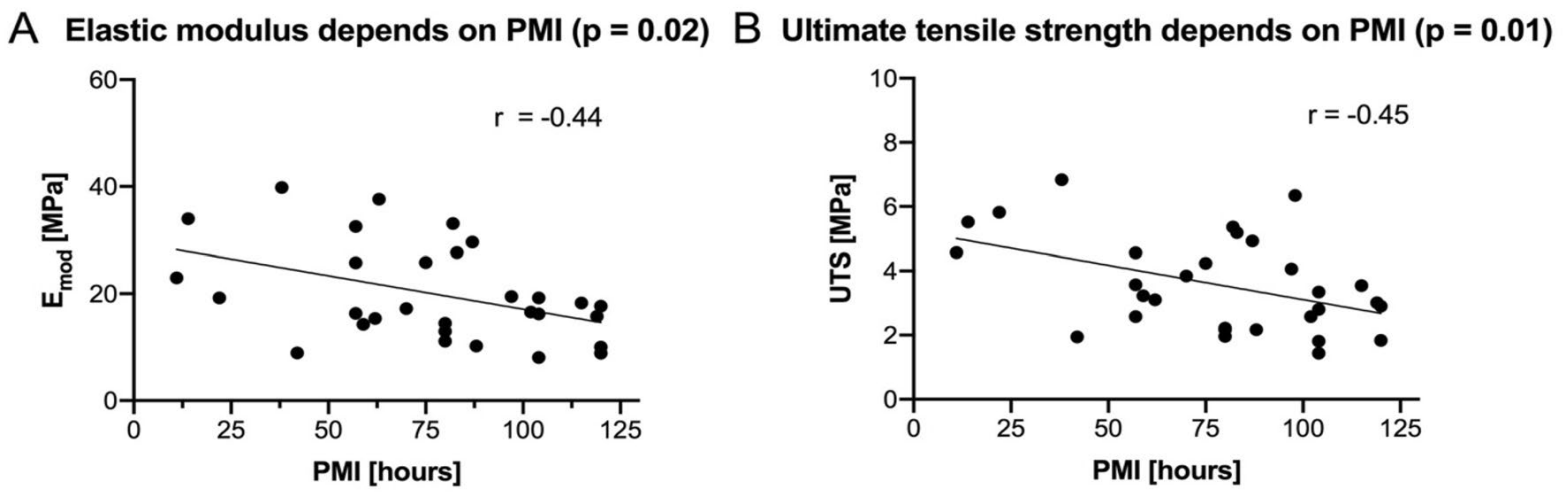
The elastic modulus (E_mod_) and ultimate tensile strength (UTS) of the human acellular scalp are depicted in relation to the post-mortem interval (PMI). (**A)** The E_mod_ reveals a moderate negative correlation with the PMI. (**B)** Equally, the UTS of the human acellular scalp negatively correlates with the PMI.

Apart from the significant correlations between PMI and both the E_mod_ as well as UTS of AS samples, no other significant correlations between biomechanical properties of AS samples and potential confounders such as age, sex and PMI were found (Fig. 3).

### Sodium dodecyl sulphate removed cellular components from human scalp samples, whereas antibody staining intensities partly varied between acellular and native scalp samples

**Figure 4:**
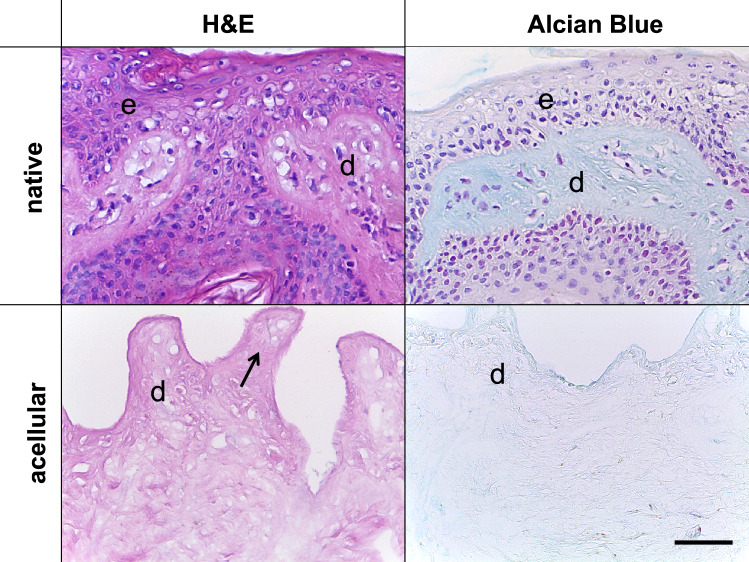
H&E and Alcian blue (AB) staining of native and acellular human scalp samples. The native scalp sample shows an epidermal (e) and dermal (d) layer in both the H&E and the AB staining. The AB stain indicates the presence of sulphated glycosaminoglycans (GAGs) in the dermal layer, represented by the light blue color. The AS sample less intensive compared to the NS sample in the AB stain. In the acellular samples, the epidermis is dissolved superficial to the dermis (D). Hence, the dermal papillae (black arrow) are superficially not covered by a cell layer after acellularization. Scale bar: 25 μm.

**Figure 5:**
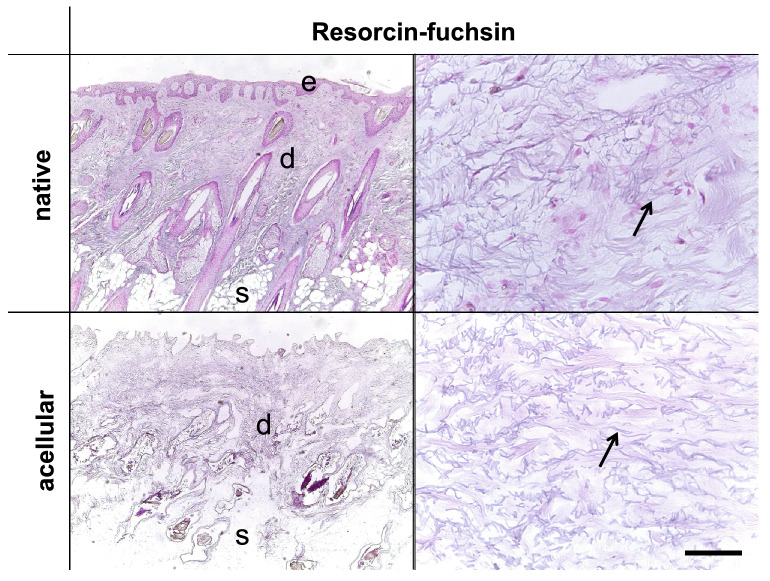
Resorcin-fuchsin staining of native and acellular human scalp samples to visualize elastic fibers. Epidermis (e), dermis (d) and subcutis (s) can be depicted in the native scalp sample. After cell removal, only the dermis (d) and subcutis (s) remained. The elastic fibers (black arrows) are clearly visible in both the native and acellular scalp samples. Cell removal appears to improve the visibility of elastic fiber’s course and structure. Scale bar: corresponds to 50 μm on the right side and 400 μm on the left side.

**Figure 6:**
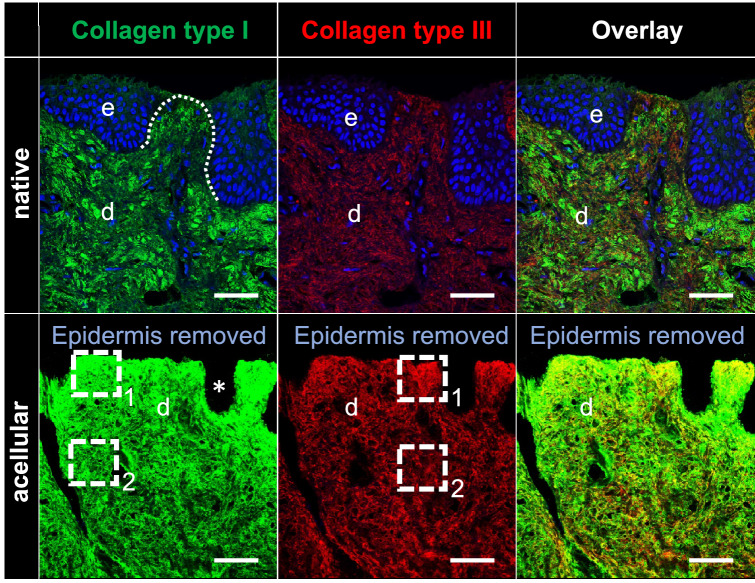
Anti-collagen-I/III immunostaining of native and acellular human scalp. Both collagen type I (green colour) and III (red colour) are present throughout the epidermis and dermis in the native samples. After acellularization, the cells cannot be depicted between the dermal papillae anymore (white asterisk) with the anti-collagen staining of acellular samples being overall more intense compared to the native samples. d, dermis; e, epidermis; white dotted line, dermal papilla; white dotted squares labeled with number 1, uniformly distributed collagen types I/III in stratum papillare of the dermis; white dotted squares labeled with number 2, dense collagen types I/III in stratum reticulare of the dermis. Scale bars: 50 μm.

**Figure 7:**
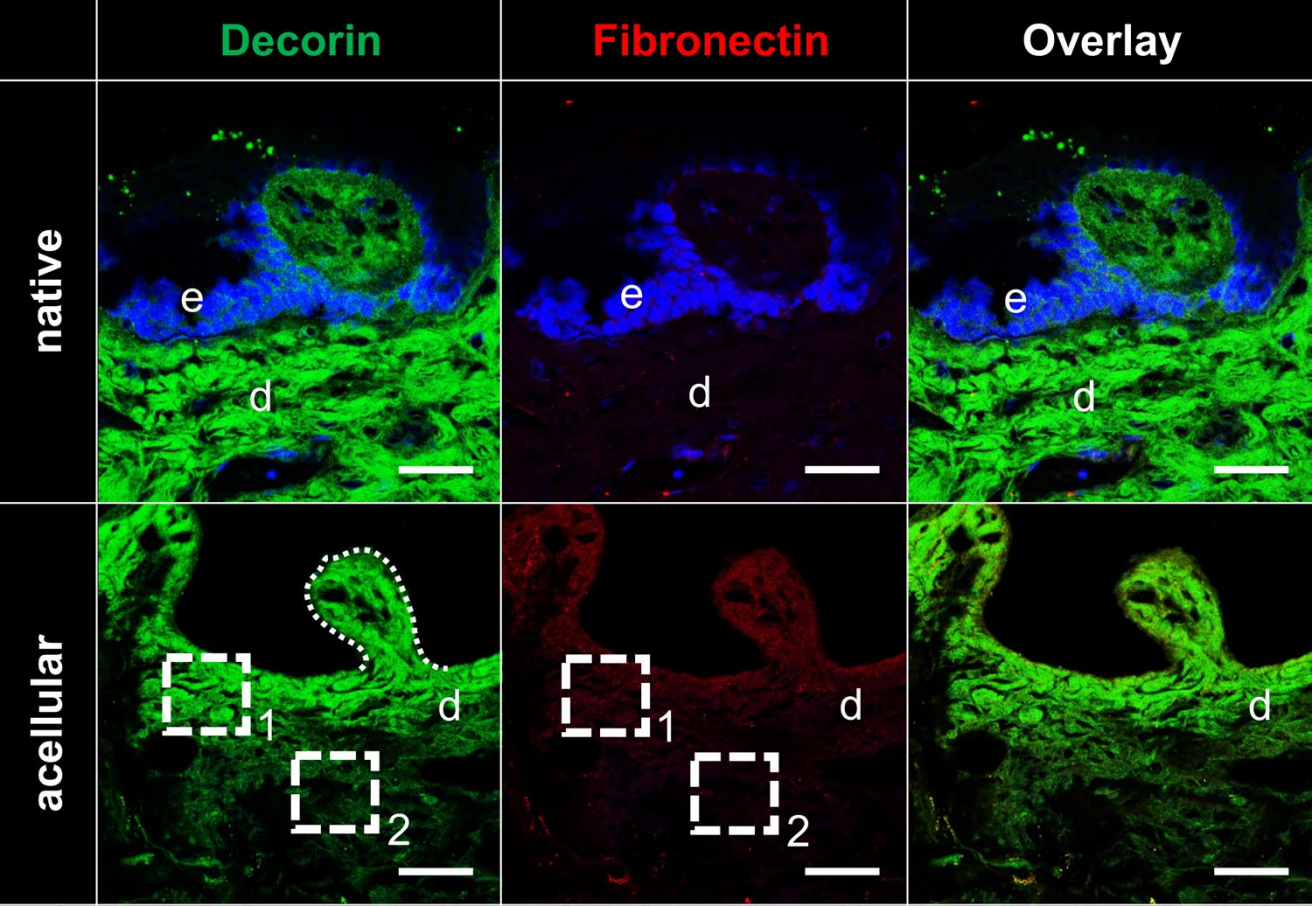
Anti-decorin and -fibronectin immunolabelings of human native and acellular scalp samples. Decorin (green color) shows a comparable green color intensity in native and acellular samples. The green color representing decorin appears to be more intense in the stratum papillare of the dermis (marked with the dotted square number 1) compared to the stratum reticulare (marked with the dotted square number 2). Fibronectin, indicated in red, is more prominent in the acellular sample compared to the native one. In line with the anti-decorin staining, anti-fibronectin staining is less intense in the stratum reticulare of the dermis (marked with the dotted square number 2) compared to the stratum papillare (marked with the dotted square number 1). Dotted line, dermal papilla; scale bars, 50 μm.

**Figure 8:**
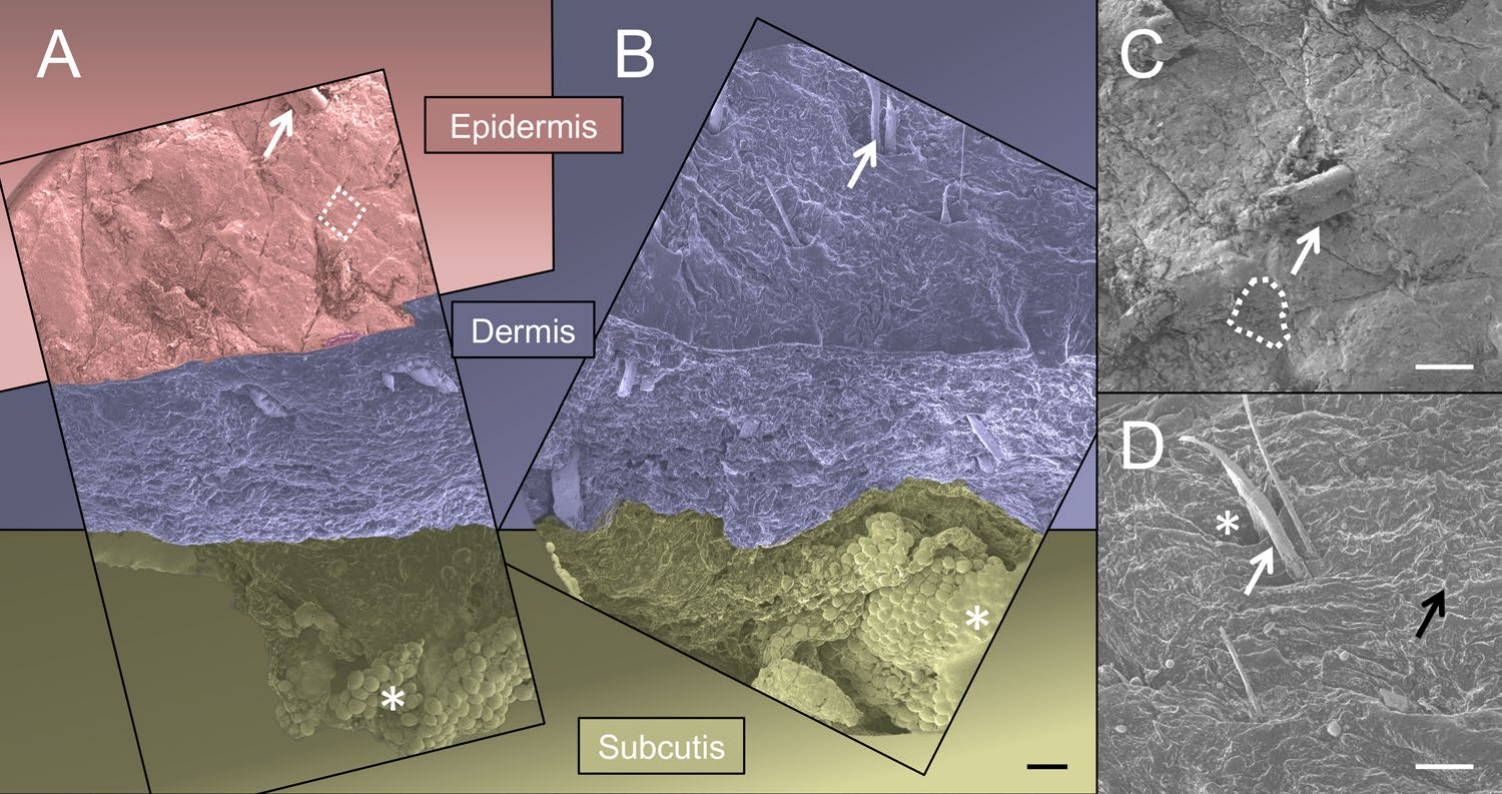
Scanning electron microscopy images of native and acellular human scalp samples. The native scalp sample **(A)** shows an epidermal (colored in red), dermal (colored in purple) and subcutaneous (colored in green) layering as expected. The white arrow indicates a hair shaft, the dotted line shows the polygonal corneocytes of the scalps’ most superficial cell layer. Subcutaneous fat is indicated by the white asterisk. In contrast, in the acellular sample **(B)** only the dermis (purple color) and subcutis (green color) can be depicted. According to A, the arrow and asterisk indicate a hair shaft and subcutaneous fat, respectively. The organization of the native scalp’s surface can be studied in greater detail in (**C)**. The white arrow points at a hair shaft and the dotted white line highlights the squamous organization of the corneocytes. The absence of the epidermis in the acellular scalp is shown in (**D)**, where the dermal papillae (black arrow). By means of acellularization, the dermal entrance to the hair follicle (white asterisk) becomes apparent. White arrow, hair shaft. Scale bars, 200 μm.

The comparison of SDS-treated NS and AS samples revealed the absence of cell nuclei and stained chromatin in the latter in the performed H&E, AB (both Fig. 4), Resorcin-fuchsin (Fig. 5), anti-collagen type I and III (Fig. 6), anti-decorin and anti-fibronectin (both Fig. 7) staining. This indicates an overall successful acellularization of the samples. Also, the SEM indicated cell removal after scalp sample treatment with SDS (Fig. 8). Acellularization appeared to weaken the AB stain (Fig. 4), but lead to more intense immunolabeling for collagens type I and III (Fig. 6) and fibronectin (Fig. 7) but not to a clear effect on decorin. In acellular samples, both the anti-collagen type I and III stainings seemed to be more prominent in the stratum papillare compared to the stratum reticulare, whereas in NS the intensity of anti-collagen staining appeared to be uniformly distributed over the different layers (Fig. 6). In the anti-collagen-I and III immunostaining as well as the anti-decorin and -fibronectin stains the native skin shows an abundance of cells, indicated by blue DAPI-stained chromatin (Figs. 6 and 7). In contrast, the absence of stained chromatin in the acellular samples depicts an encompassing acellularization with an accompanied dissolving of the epidermis (Fig. 6 and 7). Whereas collagen types I and III appear to be uniformly distributed throughout the dermal layer in the NS, they seem to be more intense in the superficial stratum papillare of the dermis (white dotted squares labeled with number 1 in Fig. 6) compared to the deeper stratum reticulare (white dotted squares labeled with number 2 in Fig. 6) in AS. After cell removal, elastic fibers seem to be visible more clearly (Fig. 5). In SEM images the superficial structure of the skin became discernable after cell removal (Fig. 8).

## Discussion

Following surgical implantation, acellular scaffolds provide cell attachment sites, thereby guiding tissue growth and remodeling^17^. The biomechanical properties of acellular scaffolds seem to be closely interconnected with these functions. This given study showed for the first time that quasi-static biomechanical properties of AS do not differ significantly from the native state when deformed in a uniaxial tensile testing environment. Likewise, the two materials do not differ regarding their capacity to withstand loads when elongated, indicating that the load-bearing collagen backbone of human skin stays intact during acellularization. A similar E_mod_ and tensile strength, when compared to the native tissue, is in line with previous investigations of our group on human iliotibial tract^2^, dura mater^12^ and temporal muscle fascia^13^. On the contrary, acellularization of porcine samples revealed decreasing values for the E_mod_ and UTS following an identical sample treatment as for the human tissues. These interspecies differences are unexpected as the porcine skin is morphologically similar to human skin and therefore frequently used as a model for the same^18,19^. The significantly higher elongation of AS compared to NS is likely a consequence of occurring ‘free spaces’ in the tissue following the cell removal and subsequent loss of many hair shafts in this study, which was proven by using histology, immunohistochemistry and SEM agreeing with previous observations^2,20^. Regarding this, the SF_max_ also increased in acellular iliotibial tract samples^2^, but stayed unaltered in human dura mater^12^, temporal muscle fascia^13^ and porcine skin^3^. The increased tissue elongation following acellularization might be a result of both the amount of occurring free spaces formerly occupied by cells and the axis of load application during the uniaxial tensile test. In human dura mater, temporal muscle fascia and iliotibial tract comparatively few cells are scattered within the predominant collagen matrix^2,12,13^. When the highly anisotropic iliotibial tract is tensile loaded in the direction of the dominant collagen orientation following cell removal, the elongation increases as collagens can fill these ‘empty spaces’. However, the increase in elongation might not be observed, when the load is applied perpendicular to the axis of the preferred collagen course. In tissues, with a more complex anisotropic organization compared to the iliotibial tract such as dura mater or temporal muscle fascia the increasing effect of acellularization on the SF_max_ might diminish due to varying collagen bundle arrangements in the loading axis of the tested sample, which cannot be detected by the naked eye, but was visualized by SEM recently^21^. Complementing this, for a tissue with a comparatively high number of cellular components in relation to the present collagens, such as the here presented human scalp, the axis of load application is less important as the occurring free spaces following acellularization are too large and consequently inevitably lead to an increased elongation of the acellular sample compared to the native one as shown in this study. All things considered, due to the different strain values of acellular skin compared to the native one, the hypothesis has to be rejected and it has to be stated that cell removal does partly influence the biomechanical parameters of human scalp.

The here mentioned ‘free spaces’ caused by the cell removal seemed to expose the epitopes recognized by anti-collagen type I and III as well as fibronectin antibodies resulting in intensified staining in the superficial skin layers, showing the decreased intensity of coloration from superficial to deep when assessed qualitatively. An increased occurrence of e.g. dermal fibronectin in proximity to the cell-rich epidermal layers seems plausible, taking into consideration that fibronectin is of importance for the cell adhesion, migration, differentiation and ingrowth of cells^22^. Removal of the unformed ECM, namely proteoglycans and glycosaminoglycans may be a further explanation for the enhanced visibility of the formed ECM, which is supported by the weakened AB staining in acellular samples. Additionally, in line with previous investigations on human dura mater^12^, acellularization does expose elastic fibers seen in standard histology and of the dermal surface in SEM. The latter could for example be used to analyze the three-dimensional architecture of dermal papillae in different body regions to study their structural adaptation to mechanical loading.

Besides their orthotopic use, acellular skin grafts are frequently used as heterotopic grafts in various body sites^5,23–25^. In this study, we have shown that human AS is significantly more elastic compared to human ATMF and ADM. When an acellular skin graft is used to cover dura mater defects, this increased elasticity of the graft compared to the original tissue should be considered, given that energy storage and dissipation might be altered compared to the original tissue^26^. This particularly applies when the graft will be used for a bridge-like duraplasty as done in surgical cranectomies^27^ without the skull limiting the expansion of the scaffold during cerebrospinal fluid pulsations. The given study showed that both AS neither differed from NS nor from compared acellular matrices (ATMF and ADM) regarding its capability to withstand loads before failing. Hypothetically, in cases of duraplasty in which the soft tissue overlying the brain is not covered by the skull^27^, a more compliant graft by being equally load-resistant such as an acellular skin scaffold could allow for greater expansions during brain edema, potentially causing less increase of the intracranial pressure^28^ and benefits the patient’s outcome after intensive care periods. ATMF and ADM did not statistically differ regarding their tensile strength from the AS scaffolds in this study but revealed differences in strain values and elastic behavior under load application. A thorough knowledge of these biomechanical cross-comparisons will lead to a large biomechanical database, which allows for an individual selection of the most suitable graft material during surgery and in addition aids as a reference for the design of new artificial 3D-printed graft materials.

To the best of our knowledge, the influence of both the donor’s age as well as the time between graft retrieval and transplanting into the recipient on the biomechanical properties of the graft were not investigated before. Our results reveal that the E_mod_ and the UTS of the human acellular skin scaffold decrease with increasing time after death. Therefore, post-mortem tissue donation should be initialized as soon as possible after the individual’s death. The decreasing E_mod_ could be caused by progressing collagen degradation or autolysis after the tissue loses vitality. This might be caused by the decreased pH value occurring in tissues, when oxygen circulation stops and therefore energy metabolism, respectively^29^.

## Limitations

The here presented study is limited by the sample size, which had been restricted by the available number of tissues for the given project. Samples in this study were harvested from the scalp region. Biomechanical acellular dermal properties of other body regions likely vary. For the performed tensile tests a quasi-static uniaxial setup was selected, which might differ from the dynamic and multiaxial properties of scalp tissues under native (‘traumatic’) conditions. Furthermore, an impact of potential skin-affecting diseases on the biomechanical parameters obtained in the given study cannot be excluded although tissues were only harvested from cadavers with no known history of systemic dermatologic disorders and tissues that were macroscopically sound in this regard.

## Conclusions

The biomechanical properties of skin samples provided in this study complement previous findings that acellularization of human scalp with SDS does not impact the scaffold’s elastic modulus and ultimate tensile strength, when compared to the native state irrespective of the origin of the tissue. The elongation, most likely dependent on the tissue’s original cell-collagen-ratio, was found to be the main biomechanical difference between native and acellular human scalp tissues. Skin grafts should be harvested as soon as possible after death to prevent degradation influencing the biomechanical properties (Supplementary information).

## Supplementary information

Supplementary Information.
