# Supplementary material for: Load-deformation characteristics of acellular human scalp: assessing tissue grafts from a material testing perspective
Source: Sci Rep. 2020 Nov 6;10:19243. doi: 10.1038/s41598-020-75875-z (PMC7648071; doi:10.1038/s41598-020-75875-z)
Supplement: Supplementary file 1 — Supplementary Information. [file 41598_2020_75875_MOESM1_ESM.docx]

**Supplementary material for paper ‘Load-deformation characteristics of acellular human scalp – assessing tissue grafts from a material testing perspective’**

Johann Zwirner^1^, Benjamin Ondruschka^2^, Mario Scholze ^3^, Gundula Schulze-Tanzil^4^ & Niels Hammer^5-7*^

1. Department of Anatomy, University of Otago, Dunedin, New Zealand
2. Institute of Legal Medicine, University of Leipzig, Leipzig, Germany
3. Institute of Materials Science and Engineering, Chemnitz University of Technology, Chemnitz, Germany
4. Department of Anatomy and Cell Biology, Paracelsus Medical University, Salzburg and Nuremberg, Germany
5. Department of Macroscopic and Clinical Anatomy, Medical University of Graz, Graz, Austria
6. Department of Orthopaedic and Trauma Surgery, University of Leipzig, Leipzig, Germany
7. Fraunhofer IWU, Dresden, Germany

**List of commercially available acellular skin grafts and their provided elastic modulus and ultimate tensile strength:**

| **Acellular skin matrix** | **Elastic modulus [MPa]** | **Ultimate tensile strength [MPa]** |
| --- | --- | --- |
| AlloDerm (LifeCell, Branchburg, NJ, USA) | 31 - 260^a,1^ | 9.4 - 21.2^a,1^ |
| AlloMax (C. R. Bard, Warwick, NY, USA) | 70^b,2^ | 14^3^ - 23^b,2^ |
| AlloMend (AlloSource, Centennial, CO, USA) | n/a | 21^4^ |
| AlloPatch HD (MTF, Edison, NJ, USA) | 44 - 74^c,5^ | 16^4^ |
| ArthroFLEX (LifeNet Health, Virginia Beach, VA, USA) | n/a | n/a |
| BellaDerm (MTF, Edison, NJ, USA) | 16^6^ | 15^6^ |
| CGderm (Daewoong Bio Inc., Seoul, Korea) | n/a | n/a |
| DermACELL (LifeNet Health, Virginia Beach, VA, USA) | n/a | n/a |
| DermaMatrix (Synthes, West Chester, PA, USA) | 8.8^4^ | 15^5^ |
| DermaPure (Tissue Regenix, Leeds, UK) | n/a | n/a |
| Epiflex (DIZG, Berlin, Germany) | n/a | 35^8^ |
| FlexHD PLIABLE (MTF, Edison, NJ, USA) | 8^7^ | 9^7^ |
| FlexHD STRUCTURAL (Ethicon, Somerville, MA, USA) | 30^b,9^ | 16^10^ |
| Glyaderm (Euro Skin Bank, Beverwijk, Netherlands) | n/a | n/a |
| GRAFTJACKET (Wright Medical Group, Memphis, TN, USA) | 55^6^ | 21^11^ |
| Karoderm (Karocell Tissue Engineering AB, Stockholm, Sweden) | n/a | n/a |
| MegaDerm (L&C BIO Inc., Seongnam, South Korea) | n/a | n/a |
| NeoForm (Mentor, Irvine, CA, USA) | n/a | n/a |
| Repliform (Boston Scientific, Marlborough, MA, USA) | n/a | n/a |
| SureDerm (HansBiomed, Seoul, South Korea) | n/a | n/a |

**Commercially available acellular dermal matrix scaffolds and their related elastic moduli and ultimate tensile strengths.** The presented values are rounded up (≥0.5) or down (<0.5) to integer depending on decimal places given in original study. ^a^ depending on hydration state, ^b^ approximated data (read from graph), ^c^ depending on graft type (HD1 or HD2), ^1^(Bottino, et al. 2009), ^2^(Annor, et al. 2012), ^3^(Deeken and Lake 2017), ^4^(CMF 2006) ^5^(Stevens and Stilwell 2014), ^6^(Barber and Aziz-Jacobo 2009), ^7^(Nilsen, et al. 2016), ^8^(Vitacolonna, et al. 2014), ^9^(Pui, et al. 2012), ^10^(Ngo, et al. 2011), ^11^(Omae, et al. 2012)

Annor, A. H., et al.

2012 Effect of enzymatic degradation on the mechanical properties of biological scaffold materials. Surg Endosc 26(10):2767-78.

Barber, F. A., and J. Aziz-Jacobo

2009 Biomechanical testing of commercially available soft-tissue augmentation materials. Arthroscopy 25(11):1233-9.

Bottino, M. C., et al.

2009 Freeze-dried acellular dermal matrix graft: effects of rehydration on physical, chemical, and mechanical properties. Dent Mater 25(9):1109-15.

CMF, Synthes

2006 DermaMatrix Acellular Dermis. Human dermal collagen matrix. West Chester, PA.

Deeken, C. R., and S. P. Lake

2017 Mechanical properties of the abdominal wall and biomaterials utilized for hernia repair. J Mech Behav Biomed Mater 74:411-427.

Ngo, M. D., et al.

2011 Evaluation of human acellular dermis versus porcine acellular dermis in an in vivo model for incisional hernia repair. Cell Tissue Bank 12(2):135-45.

Nilsen, T. J., et al.

2016 Do Processing Methods Make a Difference in Acellular Dermal Matrix Properties? Aesthet Surg J 36(suppl 2):S7-S22.

Omae, H., et al.

2012 Biomechanical effect of rotator cuff augmentation with an acellular dermal matrix graft: a cadaver study. Clin Biomech (Bristol, Avon) 27(8):789-92.

Pui, C. L., et al.

2012 Effect of repetitive loading on the mechanical properties of biological scaffold materials. J Am Coll Surg 215(2):216-28.

Stevens, P.J., and R. Stilwell

2014 The Biomechanics of AlloMend Acellular Dermal Matrix: Ultimate Tensile Strength. Pp. 7. Contennial: AlloSource.

Vitacolonna, M., et al.

2014 Effect on the tensile strength of human acellular dermis (Epiflex(R)) of in-vitro incubation simulating an open abdomen setting. BMC Surg 14:7.
